# Supplementary figures and images for: Broad-Based Influenza-Specific CD8+ T Cell Response without the Typical Immunodominance Hierarchy and Its Potential Implication
Source: Viruses. 2021 Jun 5;13(6):1080. doi: 10.3390/v13061080 (PMC8229067; doi:10.3390/v13061080)

Figure S1.

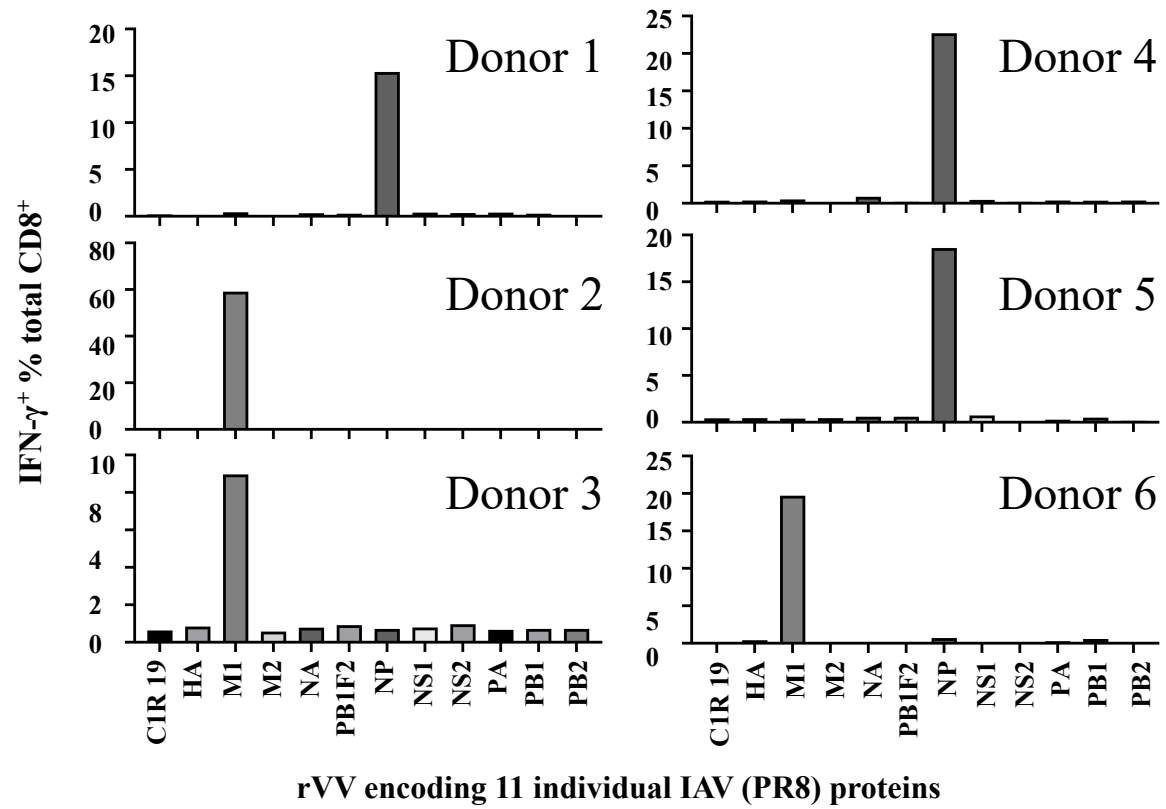

Figure S2.

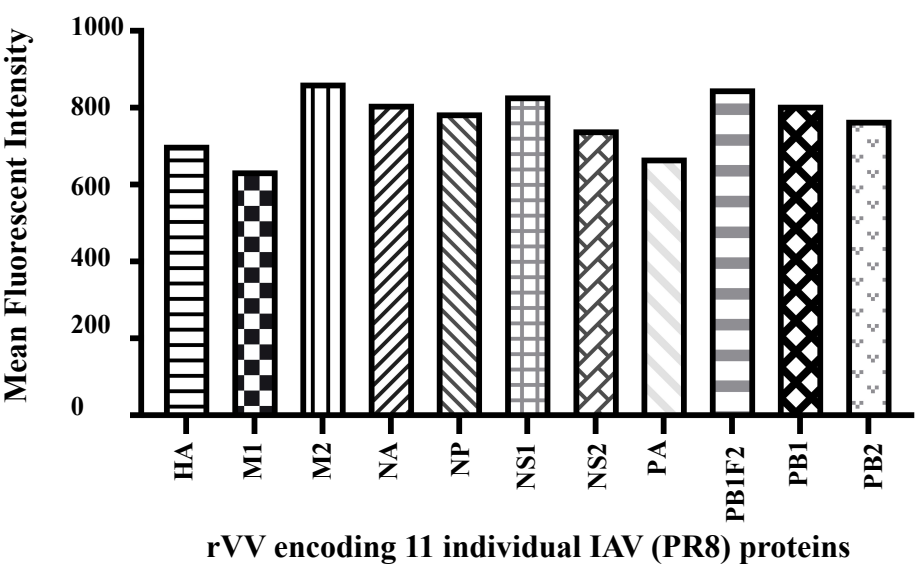

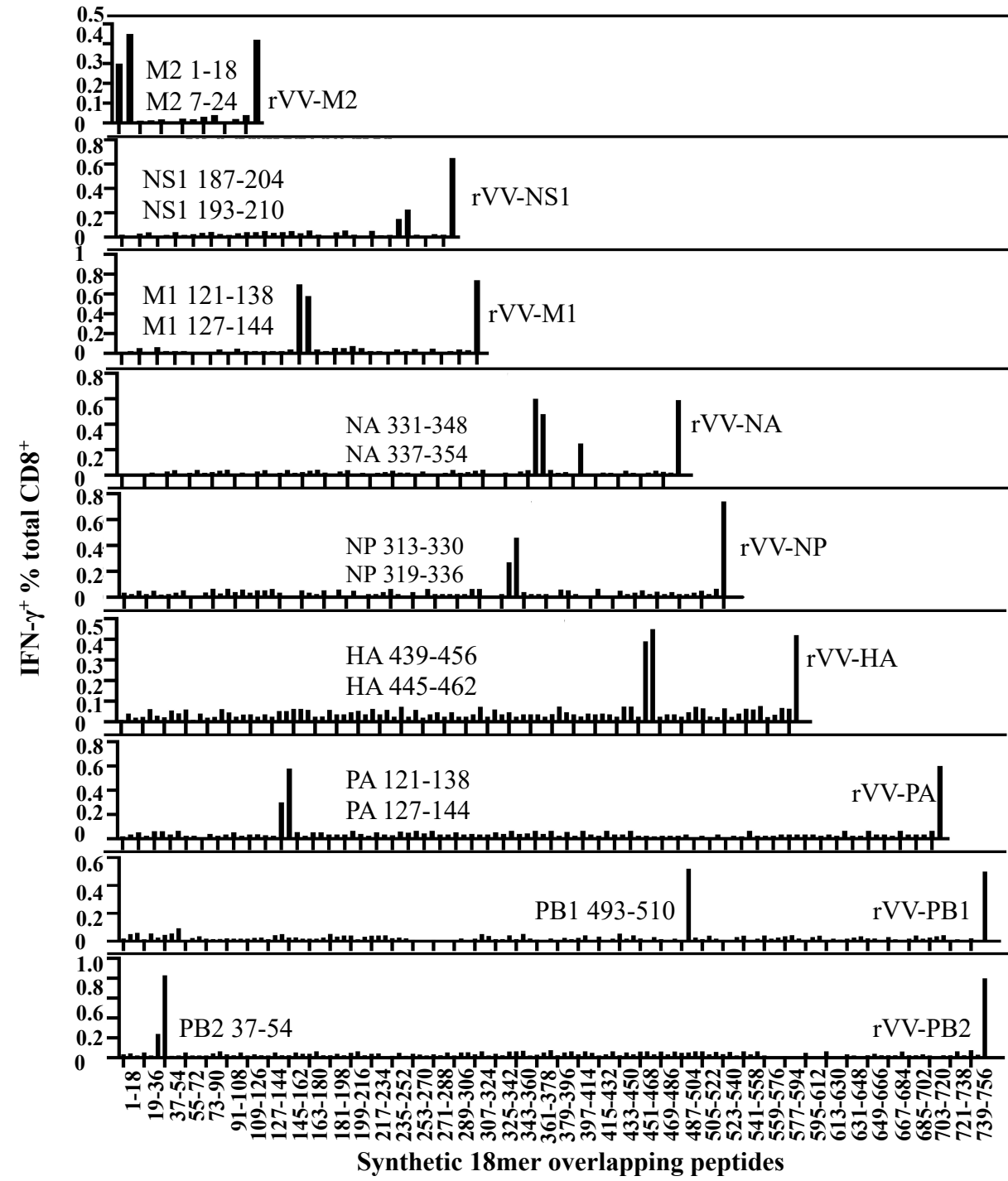

Figure S3.

Figure S4.

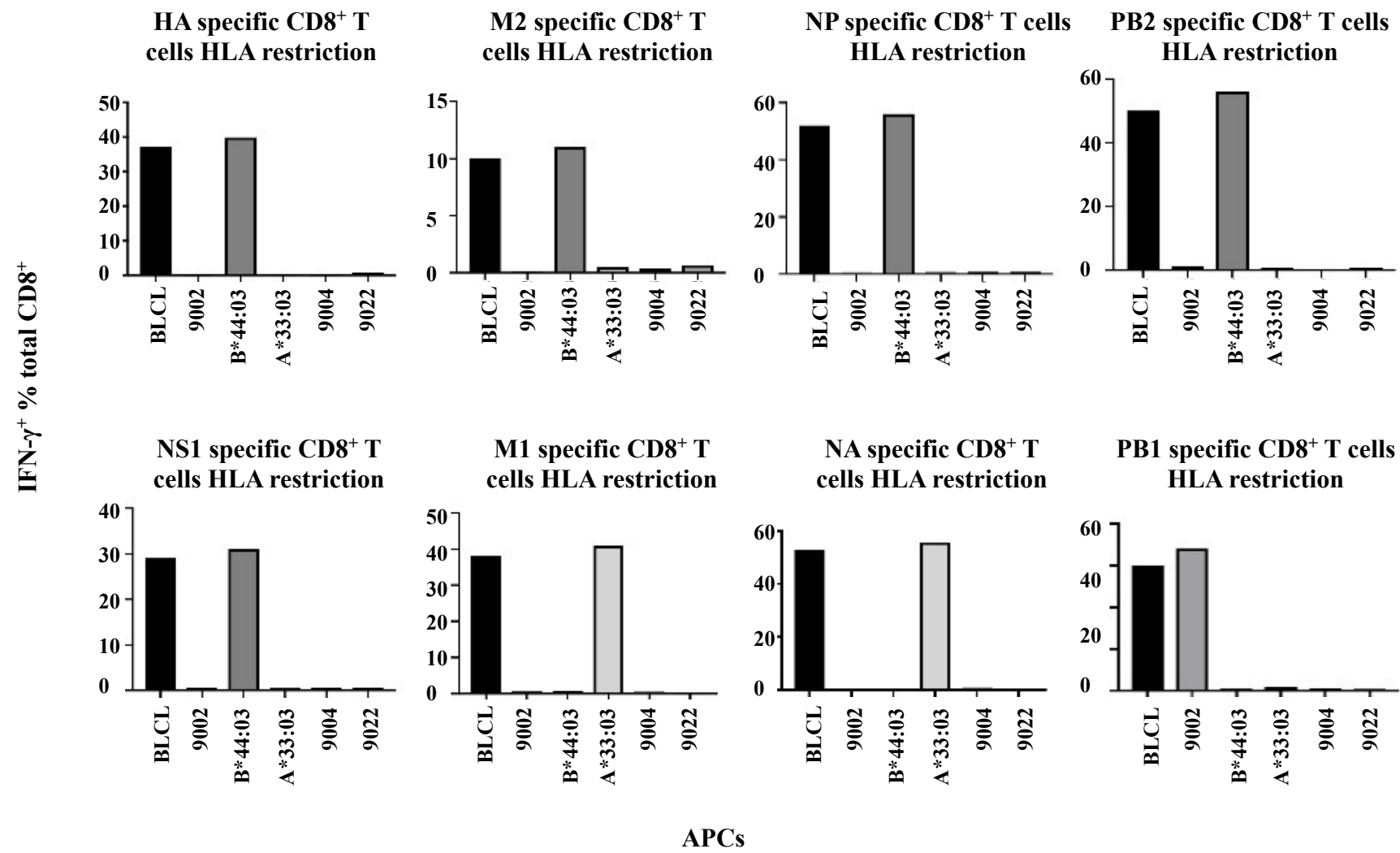

Supplement: Supplementary file 1 [file viruses-13-01080-s001.zip › viruses-1222310-supplementary.pdf]
